# Supplementary material for: Development of a Flex-Seq SNP panel for raspberry (Rubus idaeus L.) and validation through linkage map construction and identification of QTL for several traits of agronomic importance to raspberry breeding
Source: PLoS One. 2026 Feb 17;21(2):e0328606. doi: 10.1371/journal.pone.0328606 (PMC12912553; doi:10.1371/journal.pone.0328606)
Supplement: S1 File — (DOCX) [file pone.0328606.s001.docx]

**S1 File**. The 27 red raspberry varieties included in the diversity panel in this investigation.

| **Breeding code** | **Variety name** |
| --- | --- |
| SO238 | Autumn Bliss |
| SO240 | Erika |
| SO243 | Polana |
| SO244 | Polka |
| SO245 | Rossana |
| SO247 | Rubyfall |
| SO337 | Glen Lyon |
| SO350 | Cuthbert |
| SO352 | Jewel |
| SO353 | Munger |
| SO354 | Paris |
| SO355 | Milton |
| SO358 | Canby |
| SO359 | Lampone di Peveragno |
| SO360 | Latham |
| SO361 | Mammoth Red |
| SO362 | Phyllis King |
| SO363 | Puyallup |
| SO387 | Lochness |
| SO389 | Tulameen |
| SO393 | R. phoenicolasius |
| SO446 | Enrosadira |
| SO447 | Kweli |
| SO448 | Heritage |
| SO450 | Kwanza |
| SO453 | September |
| SO006 | Glen Ample |
