# Supplementary material for: Development of a Flex-Seq SNP panel for raspberry (Rubus idaeus L.) and validation through linkage map construction and identification of QTL for several traits of agronomic importance to raspberry breeding
Source: PLoS One. 2026 Feb 17;21(2):e0328606. doi: 10.1371/journal.pone.0328606 (PMC12912553; doi:10.1371/journal.pone.0328606)
Supplement: S3 File — (DOCX) [file pone.0328606.s003.docx]

**S3 File**. Summary of sequencing reads, including the number of adapter-clipped reads received from the sequencing facility (LGC), the number of reads retained after Trimmomatic trimming, and the number of reads successfully mapped to the Malling Jewel genome.

| **Metric** | **Raw reads** | **Trimmed reads** | **Mapped reads** |
| --- | --- | --- | --- |
| Total number of reads | 898,931,435 | 820,777,711 | 816,693,446 |
| Standard deviation (SD) | 384,666 | 349,271 | 349,780 |
| Standard error of the mean (SEM) | 18,093 | 16,428 | 16,452 |
| Average number of reads per sample | 1,868,880 | 1,706,399 | 1,697,907 |
| Lowest number of reads per sample | 110,002 | 100,204 | 98,992 |
| Highest number of reads per sample | 2,608,334 | 2,376,598 | 2,378,375 |
