# Supplementary figures and images for: Development of a Flex-Seq SNP panel for raspberry (Rubus idaeus L.) and validation through linkage map construction and identification of QTL for several traits of agronomic importance to raspberry breeding
Source: PLoS One. 2026 Feb 17;21(2):e0328606. doi: 10.1371/journal.pone.0328606 (PMC12912553; doi:10.1371/journal.pone.0328606)

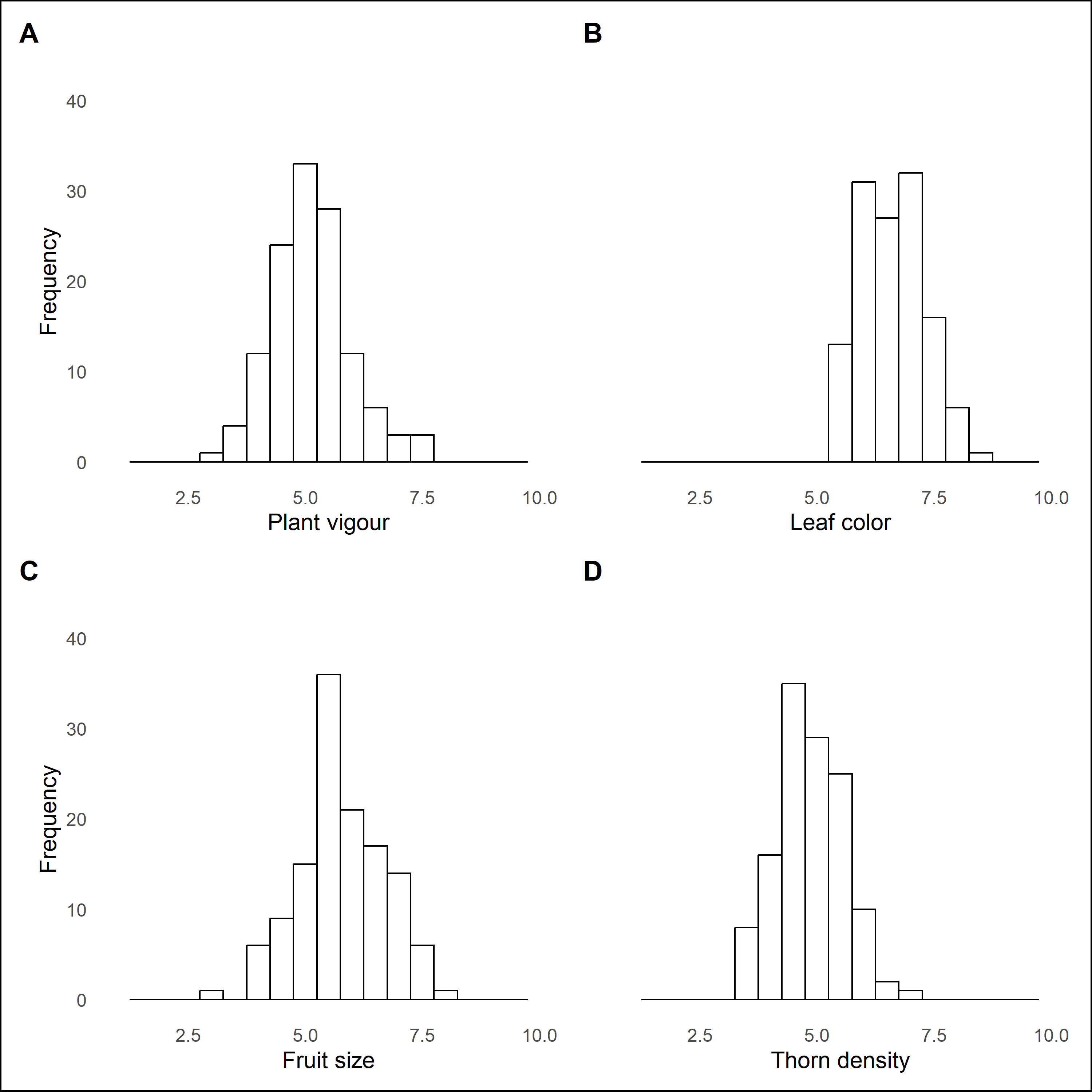

Supplement: S1 Fig — Distributions of phenotypes for (A) plant vigour, (B) leaf colour, (C) fruit size, and (D) thorn density. (TIFF) [file pone.0328606.s008.tiff]

True Neighbor-Joining (NJ) circular tree from SNP genotypes (0=AA,1=AB,2=BB)

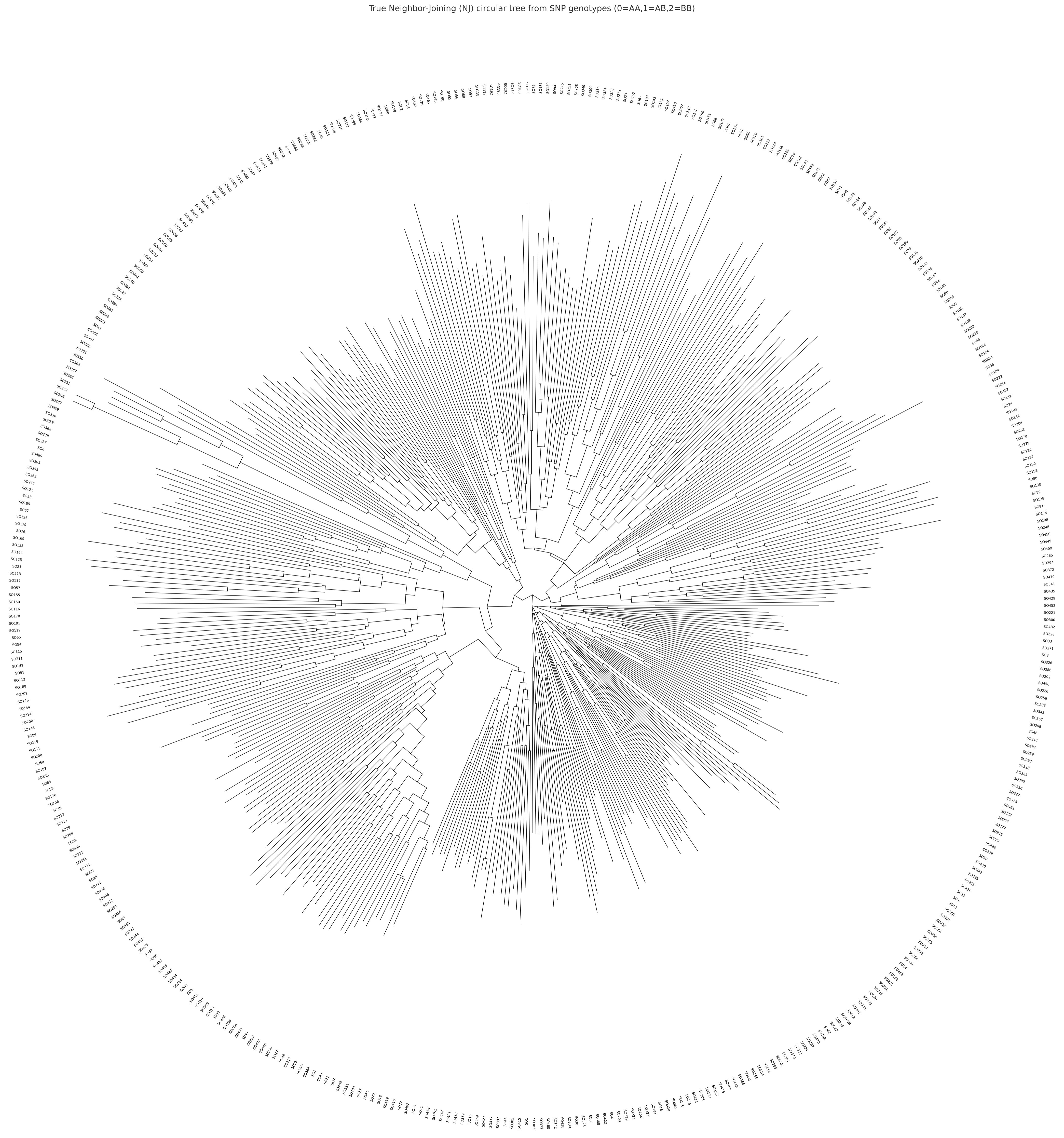

Supplement: S2 Fig — (PDF) [file pone.0328606.s009.pdf]

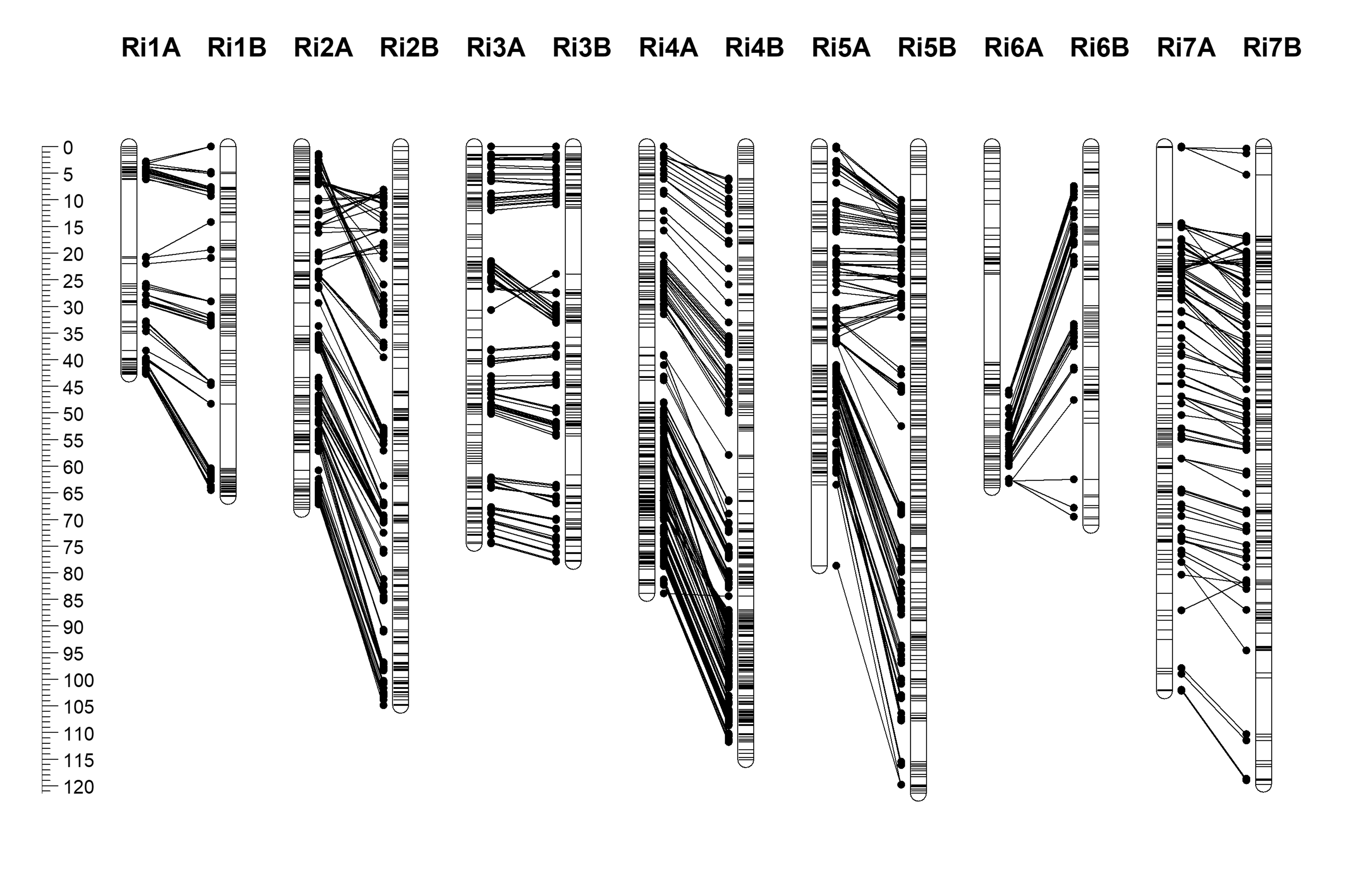

Supplement: S3 Fig — Markers segregating AB × AB that were mapped to both male and female maps are linked by a solid line between linkage maps. (PNG) [file pone.0328606.s010.png]

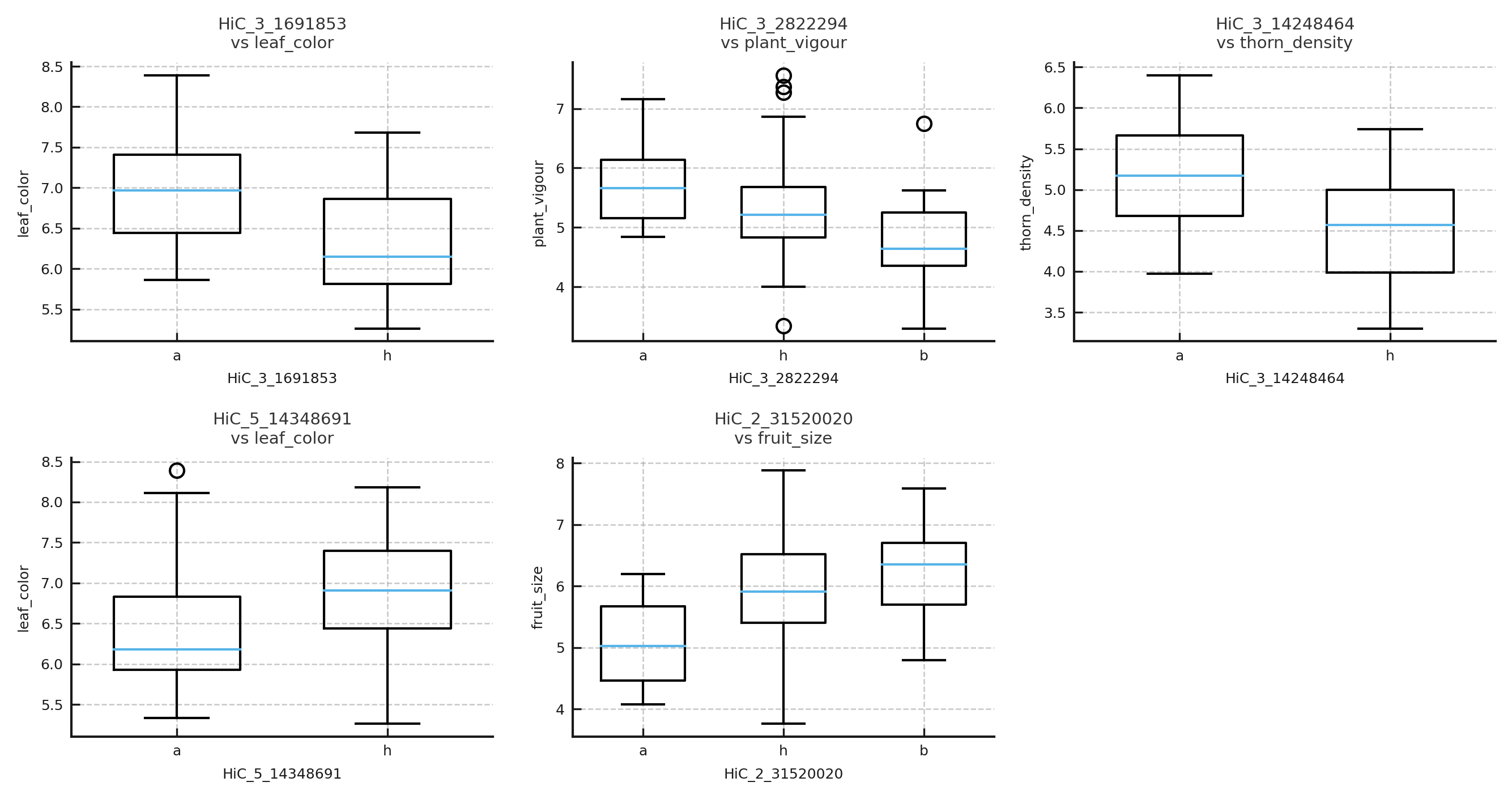

Supplement: S4 Fig — (PNG) [file pone.0328606.s011.png]

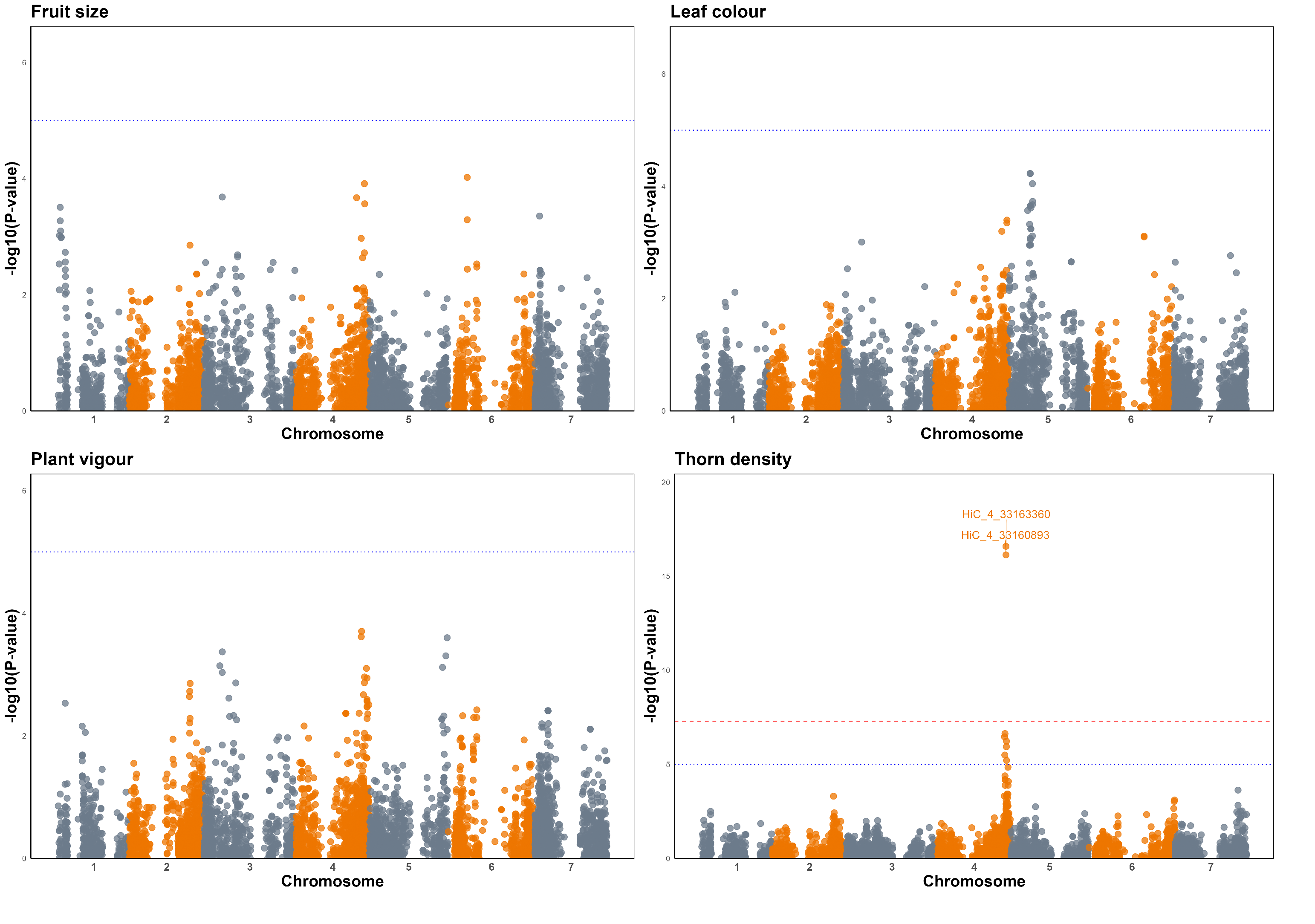

Supplement: S5 Fig — The suggestive threshold of -log10(p) ~ 5 is also indicated with the blue dotted line. (TIFF) [file pone.0328606.s012.tiff]

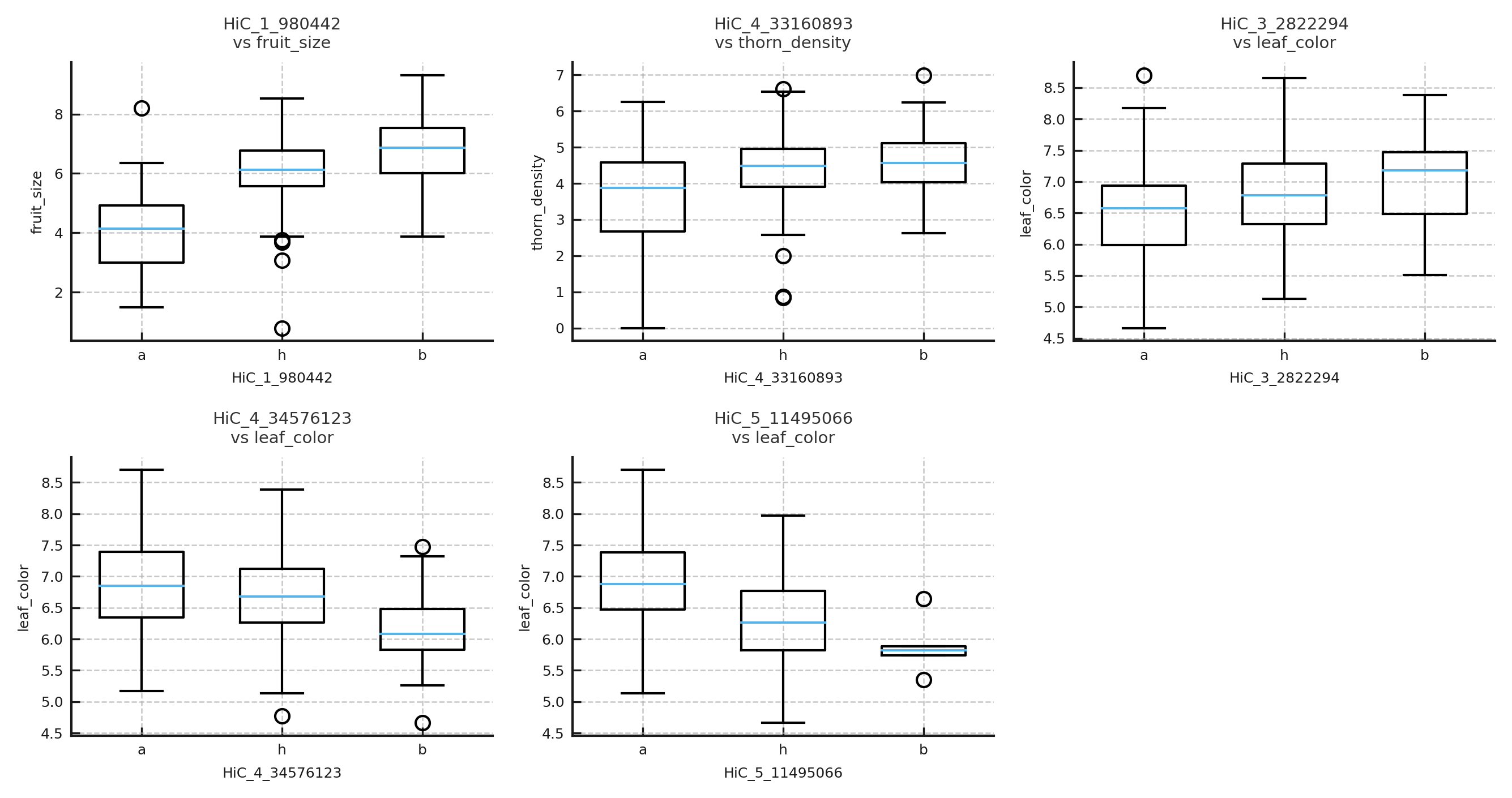

Supplement: S6 Fig — (PNG) [file pone.0328606.s013.png]

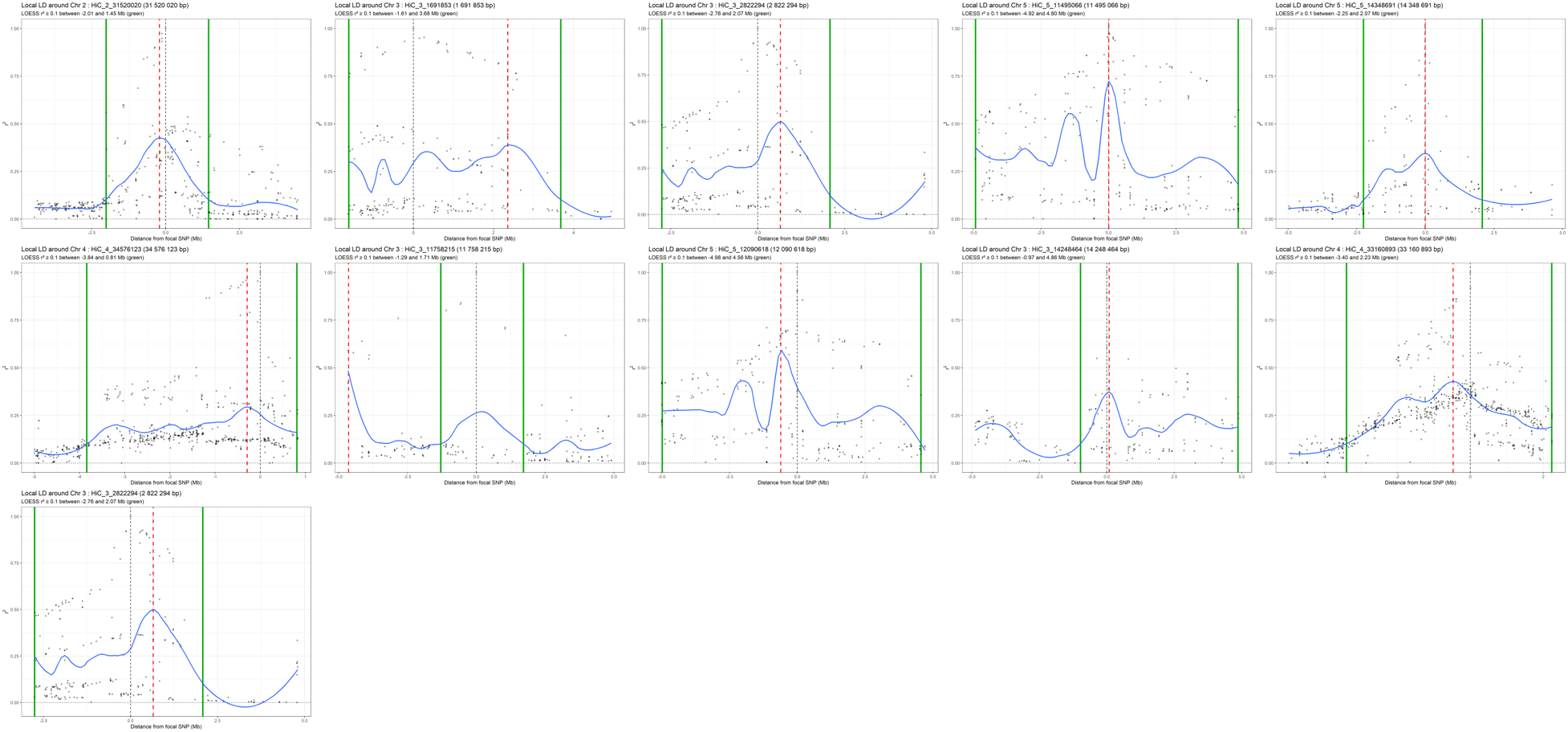

Supplement: S7 Fig — Pairwise LD (r²) was calculated between each focal SNP and surrounding markers within a ± 5 Mb window and plotted against physical distance. LOESS-smoothed curves summarise local LD decay, with vertical dashed lines indicating the focal SNP and the positions where LOESS-predicted r² drops below 0.10 (solid lines). (TIF) [file pone.0328606.s014.tif]
