# Supplementary material for: Development of a Flex-Seq SNP panel for raspberry (Rubus idaeus L.) and validation through linkage map construction and identification of QTL for several traits of agronomic importance to raspberry breeding
Source: PLoS One. 2026 Feb 17;21(2):e0328606. doi: 10.1371/journal.pone.0328606 (PMC12912553; doi:10.1371/journal.pone.0328606)
Supplement: Attachment — Submitted filename: Rebuttal_Letter_PLOS_ONE.docx [file pone.0328606.s016.docx]

Dr Jahn Davik

NIBIO

Høgskoleveien 8

N-1431 Ås, Norway

2025 09 22

Dear Professor Sassa,

We thank you and the reviewers for their thoughtful and constructive comments on our manuscript entitled

“Development of a Flex-Seq SNP panel for raspberry (Rubus idaeus L.) and validation through linkage map construction and identification of QTL for several traits of agronomic importance to raspberry breeding.”

We are grateful for the positive evaluation of our work and for the opportunity to revise our manuscript. We have carefully addressed all points raised by the reviewers, as detailed below.

# Response to Reviewer #1

**General comment:**

Flex-Seq SNP panel for raspberry (Rubus idaeus L.) is an effective resource for genetic improvement in red raspberry. QTL analysis and GWAS using the SNP panel detected several significant associations for four agronomic traits, leading to the successful identification of multiple candidate genes.

We thank the reviewer for their positive overall comments regarding our manuscript.

**Line 102:** LGC Genomics is the name of a company. We have edited the text to make this clear.

**Lines 141–142:** We appreciate the reviewer’s suggestion. The four cultivars referenced are well documented in previously published work (ref). As noted in the Methods, SNPs for the panel were derived not only from these cultivars but also from two additional mapping studies, ensuring broad representation. Because the original DNA samples were not available, we could not re-genotype them, and additional analyses such as PCA are therefore outside the scope of this study. Importantly, the effectiveness of the SNP panel is demonstrated through our QTL and GWAS results, which confirm that the set is adequate for diversity and trait mapping purposes.

**Lines 179–180:** We thank the reviewer for spotting this error. The parameters in the table were the correct ones, and the methods have been edited accordingly.

**Lines 200–203:** We have amended the text to explain our reasoning in the methods section.

**Lines 215 and 340:** This has been done. Thank you for spotting the formatting error.

**Line 231:** We have corrected to state all polymorphic markers were included. Thank you for spotting the error.

**Line 233:** We thank the reviewer for noting this omission. The MLM GWAS results have now been added to the Results section and are provided in supplementary file S5 Fig. The associations are consistent with the results obtained with BLINK, supporting the robustness of the SNP panel, however, some of the associations are below the level of significance set for the BLINK analysis.

**Lines 249–250:** High resolution versions were included in the submission and can be accessed when clicking on the links in the PDF. We have no control over the resolution in the final PDF built; this is done by PLOS. If you click on the links, you have access to the high-resolution versions, but we cannot increase the resolution in the submission as this is created automatically by PLOS from our high-resolution files.

**Line 253:** This has now been corrected.

**Lines 272 and 284:** This has now been corrected to 457, the number of genotypes studied and detailed in the methods.

**Line 275:** The format is that of a standard .BED file, but headers have been added for clarity.

**Line 278:** InDels were not considered as markers further in the study. This has now been explained in the text.

**Lines 303–304:** A possible explanation for the discrepancy has been provided in the text.

**Lines 314–321:** This has now been explained in the results section “Paris×486 Linkage map construction”.

**Line 331:** We thank the reviewer for spotting this inconsistency. SO has been removed from its occurrences in the text.

**Line 363:** We apologise for this omission. The S6 File is included with this revised submission. In addition, we have expanded the Methods section to describe the candidate gene identification approach.

**Data availability:** We thank the reviewer for spotting the omission of the sentence detailing where the reads are deposited. The raw reads from the FlexSeq genotyping were deposited in the ArrayExpress repository at EMBL-EBI (www.ebi.ac.uk/arrayexpress) and will be released upon acceptance of the manuscript for publication.

# Response to Reviewer #2

**General comment:**

This study created a new Flexseq panel for red raspberries and confirmed its effectiveness through linkage analysis, QTL analysis, and GWAS. While this paper appears to be scientifically sound, I recommend revising it prior to publication to enhance reader understanding.

We thank the reviewer for their generally positive comments, they are greatly appreciated.

## Major comment

**Present histograms or box plots for allele effects at QTL and GWAS loci.** We have now included boxplots illustrating allele effects at significant QTL and GWAS loci (Supplementary Figures Sx–Sy). These plots provide a visual representation of the effect sizes and enhance the interpretability of the results.

**Consider including LD plots for GWAS candidate gene regions.** We thank the reviewer for this valuable suggestion. However, as this study is primarily focused on the development and demonstration of a broadly useful SNP panel rather than an in-depth dissection of specific trait loci, we feel that detailed LD analyses are beyond the scope of the present manuscript. Our intention is to show that the panel can successfully identify trait associations, and we hope that the community will apply it for deeper genetic analyses, including LD mapping.

**Show the population structure used in GWAS (Admixture, PCA, or phylogenetic tree).** A NJ tree has been constructed and is included in the manuscript as a Supplementary File.

## Minor comments

**Line 271 - “150bp” should be “150 bp”** Corrected

**Line 273 - say “VCF” rather than “.vcf file”** Corrected

We trust that these revisions fully address the reviewers’ concerns and improve the clarity and impact of our manuscript. We thank the reviewers once again for their time and constructive feedback, and we hope you will now find our manuscript suitable for publication in PLOS ONE.

Sincerely,

Jahn Davik

Norwegian Institute of Bioeconomy Research (NIBIO)

jahn.davik@nibio.no
