# Supplementary material for: Development of a Flex-Seq SNP panel for raspberry (Rubus idaeus L.) and validation through linkage map construction and identification of QTL for several traits of agronomic importance to raspberry breeding
Source: PLoS One. 2026 Feb 17;21(2):e0328606. doi: 10.1371/journal.pone.0328606 (PMC12912553; doi:10.1371/journal.pone.0328606)
Supplement: Attachment — Submitted filename: Rebuttal_Letter_PLOS_ONE_auresp_2.docx [file pone.0328606.s017.docx]

Dr Jahn Davik

NIBIO

Høgskoleveien 8

N-1431 Ås, Norway

2025 12 15

Dear Professor Sassa,

We thank you and the reviewers for their thoughtful and constructive comments on our manuscript entitled

“Development of a Flex-Seq SNP panel for raspberry (*Rubus idaeus* L.) and validation through linkage map construction and identification of QTL for several traits of agronomic importance to raspberry breeding.”

We are grateful for the positive evaluation of our work and for the opportunity to revise our manuscript. We have carefully addressed all points raised by the reviewers, as detailed below.

# Response to Reviewer #1

# No revision requested.

# Response to Reviewer #2

## Major comment

While the authors state that LD analysis is out of scope, this study includes analysis of candidate genes associated with GWAS peaks. I find the analytical workflow inappropriate in the following respects: The authors extracted 2 Mbp around the peak SNP, but the reason for this 2 Mbp range is unclear. If there is a reference that can be cited, please provide it.
Generally, the correspondence between physical distance and genetic distance varies depending on chromosomal region such as whether the region is near a telomere or a centromere. In such cases, uniformly narrowing the range to a fixed 2 Mbp is inappropriate both experimentally and analytically. In this sense, if presenting information on related genes, it is crucial to properly verify indicators like LD. Furthermore, despite the primary objective being to investigate whether associations with traits can be established, please state, within the Candidate Gene Identification section, the rational justification for performing analysis on the candidate genes of SNP peaks.

**Authors’ response**:

We thank the reviewer for reiterating this point.

We have addressed the LD issue by creating LD decay plots around the most significantly trait-associated SNPs from the linkage and the GWAS analyses. These plots are added in **S7 Fig**. Analyses were restricted to ±5 Mb around the focal marker and we used r^2^ between pairwise markers and LOESS smoothing to summarize local LD patterns around. We set non-negligible LD as the distance on either side of the focal SNP at which the LOESS-predicted r^2^ fell below 0.10. The sizes of the LD certainly did vary. However, all the suggested candidate genes were located well within the proposed LD windows.

We trust that these revisions fully address the reviewer’s concerns and improve the clarity and impact of our manuscript. We thank the reviewers once again for their time and constructive feedback, and we hope you will now find our manuscript suitable for publication in PLOS ONE.

Sincerely,

Jahn Davik

Norwegian Institute of Bioeconomy Research (NIBIO)

jahn.davik@nibio.no
